# Changes in alcohol use during hepatitis C treatment in persons who inject drugs

**DOI:** 10.1111/jvh.13737

**Published:** 2022-09-02

**Authors:** Madhuri Martin, Prerana J. Roth, Jiajing Niu, Irene Pericot‐Valverde, Moonseong Heo, Akhila Padi, Brianna L. Norton, Matthew J. Akiyama, Alain H. Litwin

**Affiliations:** ^1^ University of South Carolina School of Medicine Greenville South Carolina USA; ^2^ Department of Medicine Prisma Health‐Upstate Greenville South Carolina USA; ^3^ Clemson University School of Health Research Greenville South Carolina USA; ^4^ School of Mathematical and Statistical Science Clemson University Clemson South Carolina USA; ^5^ Department of Public Health Science Clemson University Clemson South Carolina USA; ^6^ Albert Einstein College of Medicine Bronx New York USA

**Keywords:** alcohol use, directly observed therapy, hepatitis C, opioid agonist therapy, persons who inject drugs

## Abstract

People who inject drugs (PWID) are a vulnerable population at high risk for acquiring hepatitis C virus (HCV) and frequently suffer from comorbid alcohol use. This study examines the characteristics and correlates of alcohol use among study participants, the association between alcohol consumption and sustained virologic response (SVR) in patients receiving HCV treatment, changes in drinking behaviours during HCV treatment and associations of drinking over time with specific models of HCV treatment. Participants were 150 PWID with HCV who were receiving opioid agonist therapy (OAT) and enrolled in a randomized clinical trial exploring the effectiveness of three models of care for HCV treatment. The addiction severity index was the primary measure of alcohol consumption. Days of alcohol intake were evaluated longitudinally and across three treatment groups. At baseline, 31% (47/150) reported having at least one drink in the last 30 days including 24% (36/150) who reported drinking to intoxication in the last 30 days. There was no difference in SVR rates between groups. There was a significant decrease in overall days of drinking from baseline (7.78 ± 7.86) to follow‐up at Week 24 (5.78 ± 8.83) (*p* = 0.041), but there were no significant changes among those who drank to intoxication; modified directly observed therapy (mDOT) was the only group with a significant decline in days of alcohol consumption (*p* = 0.041). In this cohort of PWID on OAT, baseline alcohol consumption did not affect SVR rates. HCV treatment was overall associated with decreased alcohol consumption. In particular, mDOT was associated with decreased alcohol consumption. Given the additive effect of alcohol and HCV on the development of cirrhosis, studies should be done to investigate the complimentary effects of the mDOT model of care on alcohol cessation.

**Abbreviations**
ACASI: audio computer‐based self‐interview
ASI: Addiction Severity Index
AUDIT: Alcohol Use Disorders Identification Test
DAA: direct acting antivirals
GT: group therapy
HCV: hepatitis C virus
mDOT: modified directly observed therapy
OAT: opioid agonist therapy
OTP: opioid treatment programme
PWID: People who inject drugs
RCT: randomized clinical trial
SIT: self‐administered individual treatment
SVR: sustained virologic response

## 1 INTRODUCTION

The burden of hepatitis C virus infection (HCV) remains disproportionately high among people who inject drugs (PWID). According to a 2017 review, an overall estimated prevalence of HCV among PWID in the United States was 53%.^1^ Alcohol use concurrent with HCV infection accelerates fibrosis progression and can worsen all‐cause mortality.^2^ There is a wide range of rates of alcohol consumption in PWID with HCV diagnosis. In the German Hepatitis C Registry, 17.9% of people with HCV on opioid agonist therapy (OAT) reported alcohol use.^3^ In a multi‐centre international study of people with HCV and either recent injection drug use or on OAT, 69% reported alcohol use.^4^ HCV and alcohol use among PWID remain common; optimizing treatment for both issues is high priority.

Historically, individuals infected with HCV with concurrent drug and alcohol use were excluded from HCV treatment due to concerns for poor adherence, high cost of treatment and risk of re‐infection.^5^ The impact of alcohol use on adherence is an area of ongoing investigation. The most recent guidelines (HCV)^6^ state that baseline drug and alcohol use do not affect adherence, and patients should not be excluded from HCV treatment.^7^ Several studies in non‐PWID populations demonstrate that alcohol use is not associated with decreased SVR.^8^, ^9^ However, there are limited studies examining whether alcohol use decreases SVR in PWID.

Studies examining alcohol use during HCV treatment for PWID (with and without alcohol‐specific interventions) are limited.^10^, ^11^, ^12^, ^13^ To date, results are variable regarding both the type of interventions used and their impact on alcohol consumption. Data for interventions in the current era of direct‐acting antivirals (DAA) therapy are even more limited. In a small study offering HCV treatment to patients receiving OAT published by Watson et al., a brief intervention regarding alcohol use resulted in a 3.1 grams per day decline in alcohol consumption.^13^ However, a multi‐centre international study describing patterns of injection drug use and alcohol use showed no change in alcohol use during HCV treatment.^14^

The aims of this study were to (1) identify the baseline characteristics and correlates of alcohol consumption among study participants; (2) assess whether alcohol consumption is associated with decreased sustained virologic response (SVR) rates in PWID; (3) assess whether alcohol consumption changed over the course of HCV treatment among a cohort of HCV‐infected PWID on OAT; and (4) explore alcohol use among three models of HCV care delivered on‐site at the OAT programme.

## 2 METHODS

### 2.1 Parent study

This study is a secondary data analysis of a randomized clinical trial (RCT) designed to test the effectiveness of three models of HCV care (self‐administered individual treatment (SIT)), group therapy (GT) and modified directly observed therapy (mDOT) among PWID receiving opioid agonist therapy (OAT).^15^ Patients in each group received co‐located HCV treatment in combination with methadone maintenance treatment for opioid use. All patients initiating HCV treatment were advised that there was no known safe level of alcohol use, and to decrease and/or stop drinking alcohol. All patients received at least monthly drug and alcohol use counselling from on‐site substance use counsellors. Patients received HCV treatment in the form of 7‐day blister packs that were also used to monitor adherence.^16^ The blister packs included a combination of the following medications: telaprevir, pegylated interferon and ribavirin sofosbuvir, pegylated interferon and ribavirin; sofosbuvir and ribavirin; or a combination DAA regimen of sofosbuvir and simeprevir or sofosbuvir/ledipasvir. In the SIT arm, patients were given monthly blister packs by opioid treatment program (OTP) staff members during their visits at the methadone clinic. In the GT arm, patients had weekly HCV support group meetings during which they received their blister packs. In the mDOT arm, patients had select ‘observed’ doses of their HCV medication that were taken during their methadone treatment visits (4–6 times per week). In addition to treatment visits, each patient had research visits every four weeks during the 12‐week treatment period, and Weeks 4, 12 and 24 in the follow‐up period.^16^ The research team did not provide additional alcohol‐specific interventions. This study was approved by the Institutional Review Board of Montefiore Medical Center. Participants provided written informed consent prior to participating.

### 2.2 Participants

Participants were enrolled from 3 OAT programmes in the Bronx, New York, and were followed from October 2013 to April 2017. All participants were ≥ 18 years old with genotype 1 HCV and willing to receive HCV treatment at their OAT programme. Participants with decompensated cirrhosis, pregnancy and psychiatric instability were excluded from the study. A more detailed description of participants can be found in the original study.^15^

### 2.3 Measures

All participants completed multiple survey instruments at baseline and follow‐up visits using audio computer‐based self‐interview technology (ACASI). HCV outcomes and HIV co‐infection were documented based on a thorough medical chart review.

### 2.4 Socio‐demographics

Sociodemographic characteristics such as age, gender, ethnicity, race, income, education, marital status and living in a controlled environment were documented (Table 1).

**TABLE 1:** Characteristics and correlates of alcohol use in PWID using ASI at baseline

Characteristics	How many days in the past 30 days have you used any alcohol (beer, wine, liquor) 0–30 days			How many days in the past 30 days have you used alcohol to intoxication (got a buzz or felt drunk) 0–30 days		
	0 day	≥1 day	*p*‐value	0 day	≥1 day	*p*‐value
	(*N* = 103)	(*N* = 47)		(*N* = 114)	(*N* = 36)	
	mean ± SD/N	mean ± SD/N		mean ± SD/N	mean ± SD/N	
	(%)	(%)		(%)	(%)	
Age	51.18 ± 11.08	51.43 ± 9.75	0.893	51.89 ± 10.70	49.25 ± 10.38	0.191
Ethnicity			0.409			0.781
Non‐Hispanic	35 (34.0)	21 (42.6)		43 (37.7)	12 (33.3)	
Hispanic	68 (66.0)	27 (57.4)		71 (62.3)	24 (66.7)	
Race			0.149			0.388
African American	22 (21.4)	18 (38.3)		29 (25.4)	11 (30.6)	
Latino	61 (59.2)	23 (48.9)		65 (57.0)	19 (52.8)	
White	10 (9.7)	2 (4.3)		11 (9.6)	1 (2.8)	
Other	10 (9.7)	4 (8.5)		9 (7.9)	5 (13.9)	
Primary Language			0.297			0.501
English	71 (68.9)	37 (78.7)		80 (70.2)	28 (77.8)	
Other* (merged Spanish with other)	32 (31.1)	10 (21.3)		34 (29.8)	8 (22.2)	
Sex			0.562			0.830
Male	68 (66.0)	28 (59.6)		74 (64.9)	22 (61.1)	
Female* (merged transgender with male)	35 (34.0)	19 (40.4)		40 (35.1)	14 (38.9)	
Sex (at birth)			0.486			0.755
Male	69 (67.0)	28 (59.6)		75 (65.8)	22 (61.1)	
Female	34 (33.0)	19 (40.4)		39 (34.2)	14 (38.9)	
Marital Status			0.036			0.781
Married / living with partner	44 (42.7)	11 (23.4)		43 (37.7)	12 (33.3)	
No partner	59 (57.3)	36 (76.6)		71 (62.3)	24 (66.7)	
Education Level			0.844			0.408
Not high school grad	45 (43.7)	19 (40.4)		46 (40.4)	18 (50.0)	
High school grad	58 (56.3)	28 (59.6)		68 (59.6)	18 (50.0)	
Employment			0.851			0.971
Employed, full or part	9 (8,7)	3 (6.4)		9 (7.9)	3 (8.3)	
Retired	10 (9.7)	4 (8.5)		11 (9.6)	3 (8.3)	
Not employed	84 (81.6)	40 (85.1)		94 (82.5)	30 (83.3)	
How many days have you experienced employment problems in the past 30 days 0–30 range	6.98 ± 12.00	7.74 ± 12.62	0.728	7.21 ± 12.35	7.25 ± 11.71	0.986
How troubled or bothered have you been by these employment problems in the past 30 days			0.681			0.339
0‐not at all	6 (17.1)	1 (6.2)		7 (18.9)	0 (0)	
1 = slightly	5 (14.3)	3 (18.8)		6 (16.2)	2 (14.3)	
2 = moderately	8 (22.9)	2 (12.5)		8 (21.6)	2 (14.3)	
3 = considerably	8 (22.9)	5 (31.2)		8 (21.6)	5 (35.7)	
4 = extremely	8 (22.9)	5 (31.2)		8 (21.6)	5 (35.7)	
Homeless			0.628			0.448
No	78 (75.7)	38 (80.9)		86 (75.4)	30 (83.3)	
Yes	25 (24.3)	9 (19.1)		28 (24.6)	6 (16.7)	
Controlled environment in past 30 days			0.480			0.653
No	85 (82.5)	40 (85.1)		95 (83.3)	30 (83.3)	
Alcohol/drug treatment	11 (10.7)	6 (12.8)		12 (10.5)	5 (13.9)	
Other (Jail, Medical, Psychiatric, other)	7 (6.8)	1 (2.1)		7 (6.1)	1 (2.8)	
Live with someone with alcohol problems?			0.971			0.528
No	96 (93.2)	43 (91.5)		107 (93.9)	32 (88.9)	
Yes	7c(6.8)	4 (8.5)		7 (6.1)	4 (11.1)	
Uses non‐prescribed drugs?			1			1
No	93 (90.3)	43 (91.5)		103 (90.4)	33 (91.7)	
Yes	10 (9.7)	4 (8.5)		11v(9.6)	3 (8.3)	
AUDIT SCORE			<0.001			<0.001
Score <8	100 (0.97)	36(0.76)		110 (0.96)	26 (0.72)	
Score >/=8	3 (0.02)	11 (0.23)		4 (0.03)	10 (0.2)	
Self‐reported drug use (30 days before baseline)						
Heroin			1			0.383
0 day	84 (81.6)	38 (80.9)		95 (83.3)	27 (75.0)	
>0 day	19 (18.4)	9 (19.1)		19 (16.7)	9 (25.0)	
Other opioid/analgesics			0.228			0.466
0 day	77 (74.8)	40 (85.1)		91 (79.8)	26 (72.2)	
>0 day	26 (25.2)	7 (14.9)		23 (20.2)	10 (27.8)	
Cocaine			0.010			0.009
0 day	85 (82.5)	29 (61.7)		93 (81.6)	21 (58.3)	
>0 day	18 (17.5)	18 (38.3)		21 (18.4)	15 (41.7)	
Barbiturates			0.215			0.359
0 day	97 (94.2)	47 (100)		108 (94.7)	36 (100)	
>0 day	6 (5.8)	0 (0)		6 (5.3)	0 (0)	
Sedatives/hypnotics/tranquillisers			0.721			1
0 day	79 (76.7)	38 (80.9)		89 (78.1)	28 (77.8)	
>0 day	24 (23.3)	9 (19.1)		25 (21.9)	8 (22.2)	
Amphetamines			1			1
0 day	100 (97.1)	46 (97.9)		111 (97.4)	35 (97.2)	
>0 day	3 (2.9)	1 (2.1)		3 (2.6)	1 (2.8)	
Hallucinogens			1			0.1662
0 day	100 (97.1)	45 (95.7)		112 (98.2)	33 (91.7)	
>0 day	3 (2.9)	2 (4.3)		2 (1.8)	3 (8.3)	
Inhalants			1			1
0 day	102 (99.0)	47 (100)		113 (99.1)	36 (100)	
>0 day	1 (1.0)	0 (0)		1 (0.9)	0 (0)	
Used more than one substance at the same time			0.082			0.084
0 day	83 (80.6)	31 (66.0)		91 (79.8)	23 (63.9)	
>0 day	20 (19.4)	16 (34.0)		23 (20.2)	13 (36.1)	
Injection drug use (ever)			0.996			0.721
No	6 (5.8)	2 (4.3)		7 (6.1)	1 (2.8)	
Yes	97 (94.2)	45 (95.7)		107 (93.9)	35 (97.2)	
Cannabis (marijuana, hashish)			0.783			0.098
0 day	74 (71.8)	32 (68.1)		85 (74.6)	21 (58.3)	
>0 day	29 (28.2)	15 (31.9)		29 (25.4)	15 (41.7)	
Opiate against treatment			1			0.764
Methadone	101 (98.1)	46 (97.9)		111 (97.4)	36 (100)	
Suboxone	2 (1.9)	1 (2.1)		3 (2.6)	0 (0)	
HIV/HCV co‐infection			0.584			0.162
No	87 (84.5)	42 (89.4)		95 (83.3)	34 (94.4)	
Yes	16 (15.5)	5 (10.6)		19 (16.7)	2 (5.6)	
HCV subtype (IL2B result)			0.907			0.250
CC	23 (22.3)	10 (21.3)		22 (19.3)	11 (30.6)	
TC	55 (53.4)	24 (51.1)		64 (56.1)	15 (41.7)	
TT	25 (24.3)	13 (27.7)		28 (24.6)	10 (27.8)	
Cirrhosis			0.595			0.152
No	73 (70.9)	36 (76.6)		79 (69.3)	30 (83.3)	
Yes	30 (29.1)	11 (23.4)		35 (30.7)	6 (16.7)	
Comorbid psychiatric conditions						
ANY			0.813			0.595
No	96 (93.2)	45 (95.7)		106 (93.0)	35 (97.2)	
Yes	7 (6.8)	2 (4.3)		8 (7.0)	1 (2.8)	
Major depressive episode			0.235			0.440
No	49 (47.6)	28 (59.6)		56 (49.1)	21 (58.3)	
Yes	54 (52.4)	19 (40.4)		58 (50.9)	15 (41.7)	
Generalized anxiety disorder			0.515			0.501
No	72 (69.9)	36 (76.6)		80 (70.2)	28 (77.8)	
Yes	31 (30.1)	11 (23.4)		34 (29.8)	8 (22.2)	
Psychotic disorder			0.093			0.611
No	30 (29.1)	21 (44.7)		37 (32.5)	14 (38.9)	
Yes	73 (70.9)	26 (55.3)		77 (67.5)	22 (61.1)	
Depression (BDI)			1			0.477
None or mild ≤19	67 (65.0)	30 (63.8)		76 (66.7)	21 (58.3)	
Moderate or severe ≥20	36 (35.0)	17 (36.2)		38 (33.3)	15 (41.7)	

### 2.5 Drug use

Self‐reported drug use was assessed through the Addiction Severity Index‐Lite (ASI‐Lite; Table 1).

### 2.6 Psychiatric diagnosis

Medical charts were reviewed to obtain participants' past medical history and clinical management. The initial diagnosis was made by clinicians experienced in treating PWID; these included primary care physicians and addiction medicine specialists not directly involved with the study. Psychiatric disorders documented included depression, anxiety, psychosis, bipolar disorder, obsessive–compulsive disorder and post‐traumatic stress disorder. Additionally, the Beck Depression Inventory‐II (BDI‐II) was administered to assess severity of depressive symptoms.

### 2.7 Alcohol use

The Addiction Severity Index (ASI) measure was used to document self‐reported drinking over time. Specifically, participants were asked on how many days in the past 30 days did they drink any alcohol and how many days in the past 30 days did they drink alcohol to intoxication. Intoxication was defined as having ≥5 drinks in one day or ≥3 drinks in a sitting. The ASI was completed at baseline, at every treatment visit (every 4 weeks), and at post‐treatment follow‐up at Weeks 4, 12 and 24. Alcohol Use Disorders Identification (AUDIT) Test was not used as a continuous measure over time as AUDIT assesses drinking behaviour over the previous year as opposed to the previous 4 weeks. However, the AUDIT was used to document baseline hazardous alcohol intake defined as an AUDIT score of ≥8. Using the ASI, any alcohol drinking was defined if there had been one or more drinking days and any alcohol intoxication was defined if there had been one or more intoxicated days in the last 30 days.

## 3 DATA ANALYSIS

Baseline characteristics between participants with 0 days and ≥1 day of drinking or drinking to intoxication were compared by two‐sample t‐tests or Wilcoxon rank‐sum tests for continuous variables and chi‐squared or Fisher's exact test for categorical variables. Associations of SVR rates with any alcohol drinking days, any alcohol intoxication days and hazardous alcohol drinking (per AUDIT) were also tested by chi‐square tests. Repeated measure ANOVA was conducted to compare longitudinal alcohol consumptions during treatment period and follow‐up times between the two groups classified as above and also between three treatment groups (SIT, GT and mDOT). Time by time within groups and between groups was made using paired and two sample tests, respectively. The statistical significance was defined at *p* < 0.05. Statistical analysis was performed using R version 3.6.3.

## 4 RESULTS

### 4.1 Characteristics and correlates of alcohol use among study participants

Of the 150 participants enrolled in the study, 47 (31.3%) patients reported at least 1 day of alcohol intake in the past 30 days at baseline; 36 (24.0%) reported alcohol intake to intoxication for greater than 1 day. Using baseline AUDIT scores, 14 (9.3%) of all participants reported hazardous alcohol use (AUDIT ≥8) (Table 1). Being unmarried (*p* = 0.036) and cocaine use for ≥1 day (*p* = 0.010) were significantly associated with ≥1 day of alcohol intake in the past 30 days. Cocaine use was also associated with ≥1 day of alcohol intake to intoxication in the past 30 days (*p* = 0.009). Other substances such as opiates, barbiturates or hallucinogens did not have any correlation with alcohol use. When ASI scores were stratified as a function of AUDIT scores ≥8, people who reported >1 day of drinking (*p* < 0.001) and drinking to intoxication at baseline (*p* < 0.001) were also more likely to have AUDIT scores that met the criteria for hazardous drinking (Table 1).

### 4.2 Associations between alcohol consumption at baseline and SVR

**FIGURE 1:**
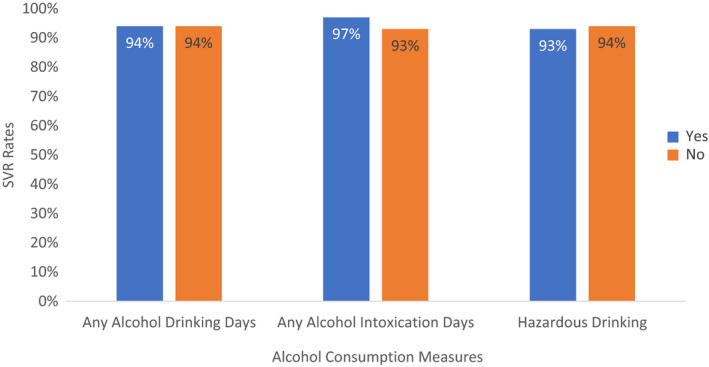
Comparison of SVR rates between groups based on alcohol consumption

Alcohol consumption at baseline was not significantly associated with SVR rates (Figure 1). Specifically, SVR rates were similar between groups of those who had any alcohol drinking days compared with those who did not (94% vs 94%, *p* = 1.000) and between those who had days of intoxication compared with those who did not (97% vs 93%, *p* = 0.595). There was also no difference between those who reported hazardous alcohol use and those who did not (93% vs 94%, *p* = 1.000).

### 4.3 Change in days of alcohol intake during treatment and follow‐up

**FIGURE 2:**
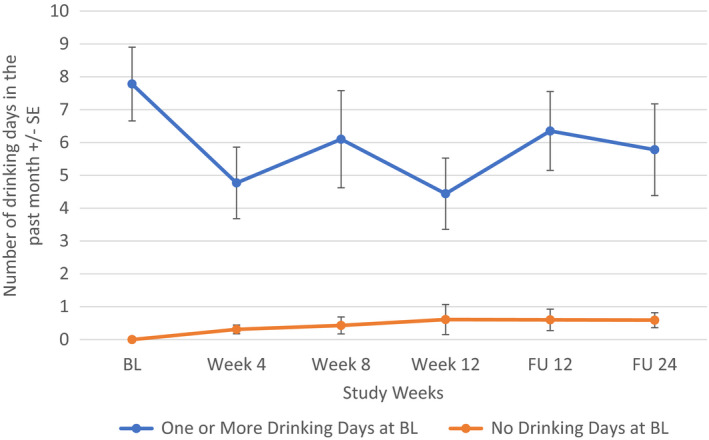
Change in days of alcohol consumption in PWID comparing groups with 0 vs ≥1 day of drinking at baseline using Repeated Measures ANOVA

When participants were grouped by 0 vs ≥1 drink at baseline, there was a decline in days of alcohol intake from baseline to follow‐up at Week 24, in patients with ≥1 day of drinking at baseline (*p* = 0.041) (Figure 2). For patients who reported alcohol use to intoxication at baseline, no significant change in alcohol use over the treatment or follow‐up periods was noted.

### 4.4 Change in days of alcohol intake as a function of assigned treatment group

**FIGURE 3:**
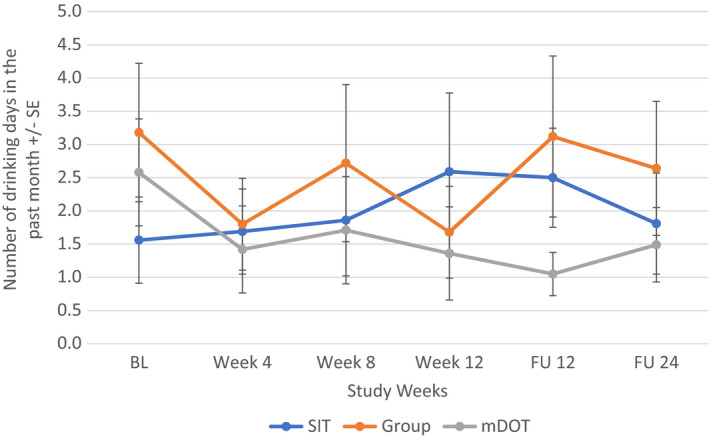
Change in days of alcohol consumption in all PWID (regardless of alcohol intake) as a function of assigned treatment group using repeated measures ANOVA

**FIGURE 4:**
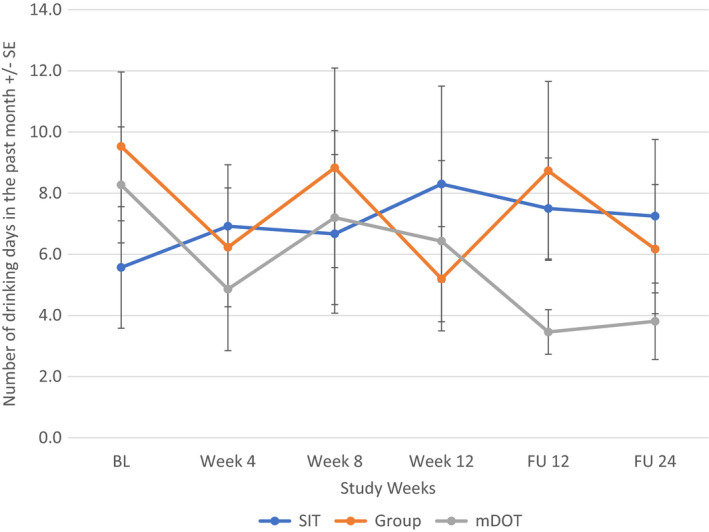
Change in days of alcohol consumption in PWID with ≥1 day of drinking at baseline as a function of treatment group using repeated measures ANOVA

**FIGURE 5:**
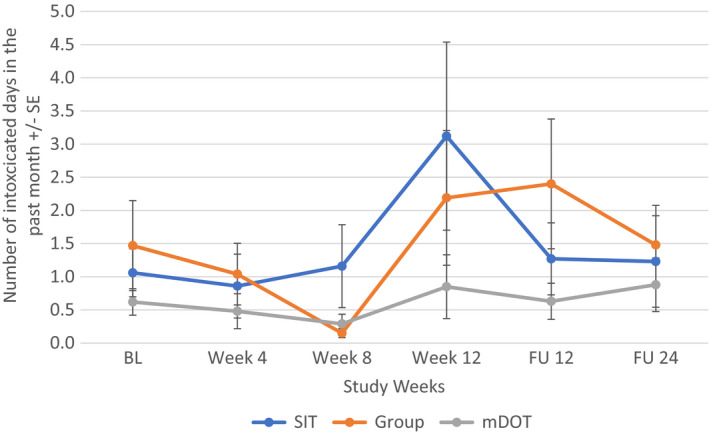
Change in days of alcohol consumption to intoxication in all PWID (regardless of alcohol intake) as a function of assigned treatment group using repeated measures ANOVA

**FIGURE 6:**
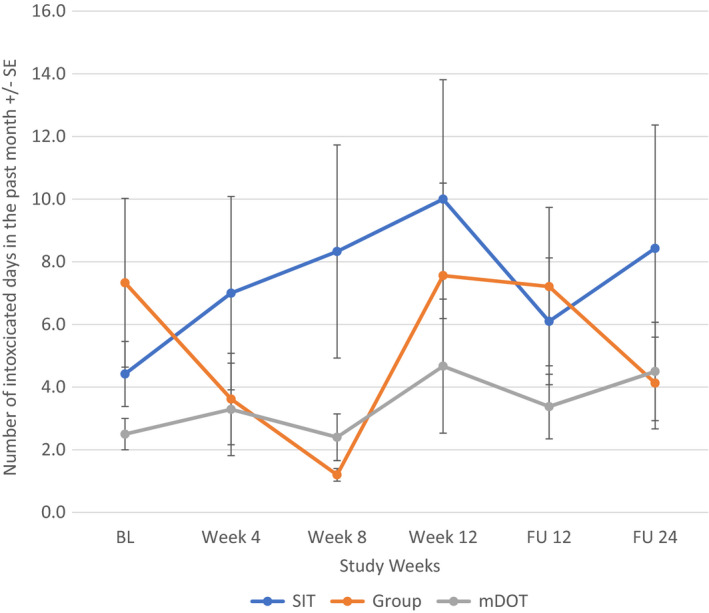
Change in days of alcohol consumption to intoxication in PWID with ≥1 day of drinking at baseline as a function of treatment group using repeated measures ANOVA

When all participants were included regardless of alcohol intake at baseline, alcohol consumption changed most significantly for the mDOT group (*F*[5, 202] = 2.666, *p* = 0.023) from baseline to follow‐up week 24: compared to the baseline level, alcohol consumption in the mDOT group at treatment weeks 4 and 12, follow‐up week 12 were significantly lower. However, alcohol consumption between any two post‐baseline time points was not significantly different (Figure 3). There were no significant changes in alcohol consumption noted in the SIT (*p* = 0.862) or GT (*p* = 0.386) treatment arm. Additionally, when looking at participants with at least one day of drinking at baseline, there was again an overall significant decrease in number of days of alcohol intake over the study period for the mDOT treatment group (*F*[5, 45] = 2.557, *p* = 0.041) (Figure 4). Treatment groups had no effect on patients with alcohol intake to intoxication at baseline (Figures 5, 6).

## 5 DISCUSSION

Our study, a secondary analysis of a RCT that offered novel HCV treatment models to PWID receiving OAT^15^ is, to our knowledge, the first to demonstrate that HCV treatment is associated with decreased alcohol use in this patient population. Given the additive effect of alcohol use and HCV on the development of cirrhosis and progression to cancer, addressing both issues is paramount. There is no known safe level of alcohol use for patients with HCV as even modest use can accelerate fibrosis.^17^ The mechanisms underlying this interaction are not fully understood but potentially include increased HCV replication, increased oxidative stress and HCV mutations and decreased host cellular immunity.^18^, ^19^

Significantly, our study demonstrates that alcohol use (any use, drinking to intoxication or hazardous drinking) is not associated with decreased SVR in PWID. This adds to existing data supporting the treatment for HCV in those who consume alcohol. A recent study did demonstrate that unhealthy drinking was associated with a higher risk of post‐treatment mortality even among those who achieved sustained virologic response.^20^ Thus, interventions to decrease alcohol use before and during treatment should be implemented.

Another significant clinical finding was a decline in number of days of self‐reported alcohol use during the study period, specifically for patients receiving mDOT therapy. This is a novel finding and should be explored further to help optimize patient health and resource utilization. Multiple factors could have contributed to the decline in alcohol use seen in the mDOT treatment model in our study. mDOT allows frequent contact with nurses, monitoring of side effects and improved adherence. Increased time with nurses may have led to the development of a trusting relationship and more time to detect alcohol use and counsel regarding cessation/referral for treatment. The PREVAIL study did not include an alcohol‐specific intervention. However, all patients received comprehensive substance use treatment at the OAT programme including illicit drugs, alcohol and tobacco use.

While exploring correlates of baseline alcohol use, our results confirm the association between cocaine use and excessive alcohol intake.^21^ Alcohol and cocaine interact to form cocaethylene, which has a longer half‐life than cocaine. Thus, many patients will drink alcohol and use cocaine together to potentiate the effect of the cocaine and ease withdrawal symptoms.^22^, ^23^

Providing HCV treatment through mDOT in patients already on OAT is a promising approach to reducing alcohol intake. Although there was no improvement in the number of days in drinking to intoxication, adding an alcohol‐specific intervention delivered by nurses while providing mDOT could improve alcohol‐related outcomes with minimal additional costs. This approach has been supported by a randomized control trial of integrated alcohol reduction in patients with HCV which offered a model of medical provider‐delivered Screening, Brief Intervention, and Referral to Treatment (SBIRT). This trial compared motivational interviewing, counselling and referral for alcohol treatment with SBIRT plus six months of integrated co‐located alcohol therapy administered by an addiction therapist. While there was no difference in treatment outcomes between the two groups, both groups showed an increased proportion of participants with full alcohol abstinence during the study. This suggests that patients with HCV infection are open to engaging in some form of alcohol treatment when encouraged to do so by their medical providers. With extending training of existing providers or adding co‐located services, this study offers models that can help reduce alcohol intake for patients undergoing HCV treatment.^24^

Limitations of our study include the use of self‐reported measures of alcohol use. We collected only frequency of alcohol use instead of both frequency and quantity which may have helped us better describe our patients' alcohol use patterns.

## 6 CONCLUSION

Alcohol use was not associated with changes in SVR rates among PWID on OAT. Alcohol use decreased overall during HCV treatment and appears to be most pronounced in the group receiving HCV treatment through mDOT. An mDOT treatment model may provide synergistic benefits to interventions targeting alcohol use in this patient population. Further research should be done to assess whether results are reproducible and cost‐effective, and whether decreased alcohol use is sustained over time.

### AUTHOR'S CONTRIBUTIONS

Madhuri Martin and Prerana Roth prepared the initial manuscript. Jiajing Niu and Moonseong Heo completed all statistical analyses. Irene Pericot‐Valverde, Brianna Norton, Matthew Akiyama, Akhila Padi and Alain Litwin substantially contributed to the editing and content of the manuscript. All authors have reviewed the final article.

## FUNDING INFORMATION

No specific funding was received for this project.

## CONFLICT OF INTEREST

Alain Litwin has served on advisory boards for Merck Pharmaceuticals, AbbVie and Gilead Sciences. Alain Litwin has received research grants from Merck Pharmaceuticals and Gilead Sciences. No other authors declared any conflict of interest related to this work.

## Data Availability

The data that support the findings of this study are available from the corresponding author upon reasonable request.
